# Predictive coding and attention in developmental cognitive neuroscience and perspectives for neurodevelopmental disorders

**DOI:** 10.1016/j.dcn.2025.101519

**Published:** 2025-01-22

**Authors:** Anne-Lise Marais, Nadege Roche-Labarbe

**Affiliations:** Normandie Univ, UNICAEN, INSERM, COMETE, GIP CYCERON, Caen 14000, France

**Keywords:** Predictive coding, Sensory prediction, Attention, Neurodevelopmental disorders, Neonatal screening

## Abstract

Sensory prediction and repetition suppression are closely related cognitive mechanisms that allow the brain to form predictions about the environment, and guide perception in synergy with attention. Predictive coding is a theory of the fundamental role of predictive mechanisms in brain functions. Authors have proposed a central role of predictive impairments in autism and possibly other neurodevelopmental disorders. However, little is known about predictive mechanisms in typical development, and how they co-develop with attention. Here we review experimental support for predictive coding and its links with attention in healthy adults’ brains, the first experimental works performed in typically developing children and infants, and theoretical accounts of neurodevelopmental disorders using a predictive coding framework. We propose future directions for predictive coding research in development. Finally, we describe the first predictive coding experiments in neonates and provide research perspectives for using this framework in searching for early markers of atypical neurodevelopment.

## Highlights

• Prediction and attention are core processes of neurodevelopment.
• Predictive coding has become a prominent framework in developmental cognitive research.
• Prediction and attention are altered in neurodevelopmental disorders and infants at risk.
• Sensory prediction has been recently explored in premature neonates.
• Prediction measures are promising biomarkers for neonatal neurodevelopmental disorder screening.

## 1 Introduction

Neurodevelopmental disorders (ND) are a group of psychiatric disorders that concern 1–17 % of the population worldwide (Francés et al., 2022) and represent a growing medical and social challenge. They are characterized by sensory, cognitive, or social impairments that lead to disabilities and school difficulties and include autism spectrum disorder (ASD), attention deficit hyperactivity disorder (ADHD), learning disorders, intellectual disability, communication disorders, and motor disorders (Crocq and Guelfi, 2015). We are still far from understanding how these disabilities emerge, hindering our ability to implement early screening and preventive interventions that would be necessary to limit high-severity symptoms. Many theories were proposed to explain ND origins, mostly for ASD (Lawson et al., 2014, Noel and Angelaki, 2023), and evidence for prenatal origins is accumulating (Bonnet-Brilhault et al., 2018), but there is no consensus to date. However, several authors have recently put forward that many of the symptoms, particularly the earliest, prodromal ones, could be explained by deficits in predicting sensory inputs (Cruys et al., 2014, Lawson et al., 2014, Pellicano and Burr, 2012, Sinha et al., 2014). This proposal is built on the predictive coding theory, a theoretical framework proposing the brain’s prediction abilities, as first formulated by H. Helmholtz, are the basis of all cognitive functions (Friston, 2005). In parallel, there has been a growing interest in predictive coding from authors working on neonatal and infant sensory processing and cognition, such that it is time to review this line of research and outline future directions for cognitive scientists working with neonates or searching for early markers of ND.

After introducing predictive coding concepts that are useful for neurodevelopmental cognitive neuroscience, we review the main experimental studies supporting this model and illustrate its mechanisms with experimental findings in adults. We provide schematic representations of the relationships between predictive coding concepts and electroencephalography (EEG) event-related potentials (ERPs). In doing so, we intend to make it easier for the reader to relate the concepts to experimental data, which to our knowledge has not been provided by previous reviews on predictive coding. This work provides a common framework for interpreting ERP data in the typical subject, but also a reference for observations in atypical or developing populations. Moreover, formalizing the correspondence between cognitive concepts and ERPs should facilitate the inclusion of future work in the integrative framework of developmental cognitive neuroscience. We feature relevant findings from other modalities such as fNIRS and fMRI, although we could not provide an exhaustive review of these modalities in the scope of this work. The long-term aim is to complete the diagrams with their atypical or immature counterparts, as well as with corresponding markers in other modalities than EEG. Then, we explore the relevance of the predictive coding framework for studying cognitive development with experimental illustrations in children and infants. We highlight the relationships between prediction and attention and emphasize the need to better distinguish between the two phenomena when designing and interpreting cognitive experiments, in both adults and children. We provide future directions for research on predictive coding in children and infants. We discuss how predictive coding impairment can contribute to the development of ND. Finally, we propose prediction as a neonatal screening marker for ND and provide suggestions for predictive coding experiments in very young subjects.

**Table:** 

Terms **Low / High order cortex:** Place of a specific cortical area in the order of sensory processing steps. The first areas processing sensory inputs are called low-order areas. Typically, they include the primary sensory cortices. High-order cortices often include the frontal areas. **Bottom-up / Top-down:** Direction of the information in the brain. Bottom-up is signal traveling from low-order cortices to high-order cortices. Top-down is the reverse. **Local / Global:** Can be said of a rule or a deviance. A local rule refers to the likelihood of an input based on adjacent (in space and/or time) inputs. A local deviance means that a stimulus differs from the adjacent ones. A global rule refers to the likelihood of an input based on the stimulation structure at higher levels (long-term patterns, or *a priori* knowledge). A global deviance means that a stimulus does not follow the over-arching structure of the sequence. **Internal model:** The representation formed of a stimulus at various levels of the processing stream, which serves to form predictions. **Prediction:** Expected input of any level of the processing stream based on the mental model of a stimulus, that serves as a basis for processing upcoming inputs. **Prediction signal:** Top-down signal communicating the prediction based on the mental model of the stimulus, resulting in a change in voltage values compared to baseline at early latencies during the omission of a predicted input. **Error signal:** Bottom-up signal indicating the mismatch between the predicted input and the actual input at any level of the processing stream, resulting in increased amplitudes at various latencies of ERPs compared with conditions that match the prediction. The difference in amplitude is called a mismatch response. **Mismatch response (MMR):** A different peak amplitude on ERPs between predicted (“frequent” or “standard”) and unpredicted (“rare” or “deviant”) stimuli. Mismatch responses can occur on multiple peaks depending on how the error signal is processed. In adults, the most reported is the ***Mismatch Negativity (MMN)*** at intermediate latencies. **Learning:** the refinement of the mental model of a sensory stimulus and of its likelihood in various contexts, through repeated exposure. **Repetition suppression (RS):** The decrease of a measure of brain activity across repetitions of a stimulus. It is sometimes called neural habituation. In fMRI studies, it is sometimes called fMRI adaptation. **Habituation:** The decrease of a measure of behavioral response across repetitions of a stimulus.

## 2 The predictive coding theory

**Fig. 1:**
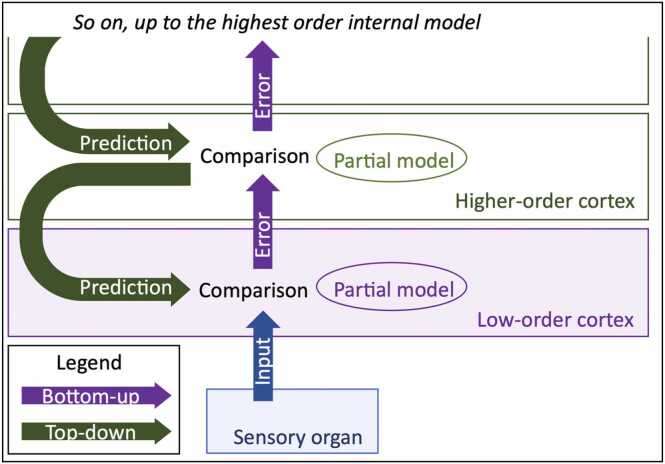
Cerebral cortices continually generate mental models of inputs from bottom-up information, and predictions about future inputs to inform (top-down information) future processing of sensory inputs. At every step, only the error between prediction and input is forwarded.

Predictive coding is a theoretical framework introducing the Bayesian statistical approach to the study of brain functions. It states that the brain continually updates mental models of sensory inputs to generate predictions about the environment (Friston, 2005) (Fig. 1).

This framework is not the first to propose that brain functions rely on comparing sensory inputs with inputs predicted on the basis of experience. The first habituation-novelty reaction experiments in infants were interpreted that way (Frantz, 1964) and yielded a long line of research on neonatal and infant learning (for a comprehensive review see Zelazo, 2023). In the scientific community of action control, authors have also attributed effective adjustment of movement and posture to the ability of the brain to anticipate sensory consequences of the movement based on previous experience (Sokolov et al., 2017, Welniarz et al., 2021). What the motor control literature named the “efference copy/forward model”, and what the infant learning literature named the “schema”, are equivalent to the concept of “prediction”. All three frameworks postulate an error-based learning process. However, Friston’s formulation proposes that the predictive process is not centralized to a specific part of the brain (that would be the locus of the internal representation being formed during learning and used for predictions), but distributed at all levels of the processing stream, with error signals being fed back to the next higher order processing step. The predictive coding view of a distributed process of prediction and error in the brain has been validated at the single-cell level in sedated and awake rodents, with evidence of error signals in subcortical regions, increasing as the signals move towards sensory cortices until the latter display a large mismatch response (MMR) (*i.e.,* a larger amplitude of neural activity when stimuli do not match the prediction) (Parras et al., 2017).

Predictive coding is appealing to developmental neuroscientists as it can integrate brain signals observed using functional neuroimaging techniques and conceptualize in a single framework the multiple neural correlates of cognitive processes from different brain areas. Because it accommodates classical concepts of developmental psychology such as schema, it provides a substrate for behavioral concepts and experimental designs in infant cognitive science. Therefore, researchers who need neural measures to go beyond the limitations of the behavioral repertoire or communication abilities of their populations of interest have seized the predictive coding theory as a promising framework. This is important for example with neonates, where motor activity can hardly be used as a proxy for cognition contrary to infants (Dooley and Blumberg, 2018), and with patients with intellectual, motor, or communication disabilities.

**Fig. 2:**
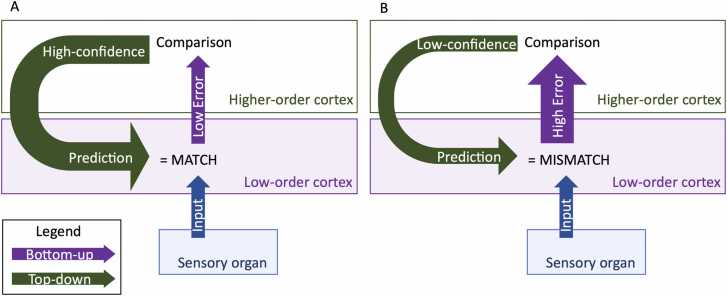
The error and the mental model are affected by the similarity between the prediction and the sensory input. When they match (A), the error is small and the confidence in the prediction is increased. When they do not match (B), the error is large and the confidence in the prediction is decreased.

Predictive coding theory describes a continuous and circular process: perceptual inputs are compared to predictions by low-order cortices; the difference (the error signal) is fed to higher-order cortices (bottom-up information), while higher-order cortices produce and update a mental model of the stimulation by learning its characteristics and generate predictions that inform lower order cortices (top-down information). When the sensory input matches the prediction, the mental model is stable, producing better predictions, and prediction confidence increases (Bubic et al., 2010) (Fig. 2). This process happens at every level. Thus, predictive coding would make sensory processing faster and less resource-consuming because inputs are not processed as new data, but as data the brain may know, and only error signals are forwarded to higher levels for processing. In addition, anticipating the most likely sensory consequences of events (including the subject’s own actions) would allow for *a priori* movement and posture adaptation, hence, more efficient behavior.

**Fig. 3:**
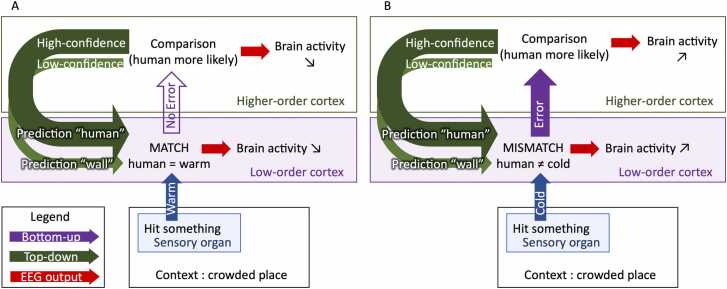
The mental model provides weighted hypotheses based on contextual information, that are used to form reliable predictions regarding future inputs. When inputs match the likely hypothesis, there is no error and brain activity measures should decrease (A). When inputs do not match the most weighted hypothesis, error transmission and model update should lead to increased brain activity measures.

Lawson et al. (2014) stated that inferring the causes of sensory inputs had two major implications. First, as multiple causes can generate a stimulus, the brain must make various hypotheses before generating a prediction: *stepping back, did I feel pressure on my back because I hit a wall or because I hit someone?* Second, the brain must attribute an amount of confidence to each hypothesis depending on prior knowledge and the context: if I am standing in a crowded bar, and the object I touched is warm and soft, another human being is more likely. The most probable hypothesis will be used in priority to form a prediction (Fig. 3). Therefore, an important challenge is to understand the relationships between various aspects of the context, both bottom-up (input reliability, or “noise”) and top-down (intentions, prior knowledge, or cues), and the various brain signals observed in predictive coding experiments. The more errors to be processed, the more brain activity should be measured.

Hereafter we review some of the works aiming at understanding the mechanisms of predictive coding and the hierarchy of brain responses reported in adults using various approaches.

## 3 Predictive coding in neurotypical adults

### 3.1 Repetition suppression, or enhancement, as a result of prediction

**Fig. 4:**
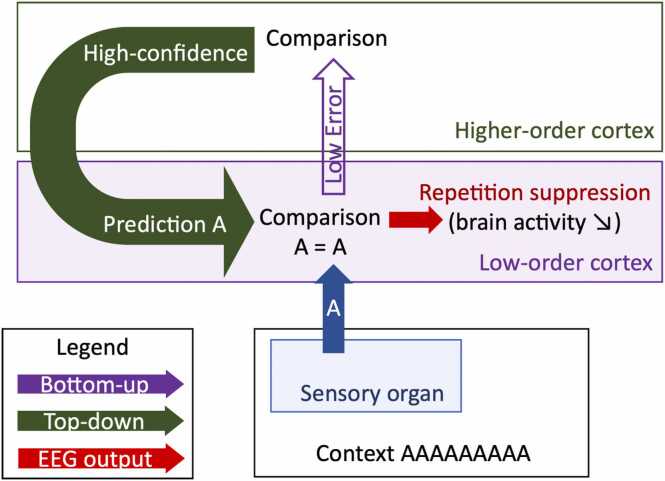
When identical inputs are repeated, the mental model generates highly reliable predictions that match inputs, and brain activity decreases across trials. This is called repetition suppression (RS).

Repetition suppression (RS) designates the reduction of a measure of brain activity across repetitions of a stimulus (Nordt et al., 2016, Summerfield et al., 2008). The phenomenon has been observed long before the predictive coding theory was formulated. It was first proposed as a passive phenomenon, neuronal fatigue, where neurons would be less activated by the stimulus across repetitions, then as sharpening, where fewer neurons would be activated across repetitions as a result of specialization, and as facilitation, where neurons would process repeated inputs more efficiently (see a review of these models in Grill-Spector et al., 2006). However, in the predictive coding framework, it came to be discussed as a result of active prediction. When there is a good match between input and prediction, and the input is not relevant to the subject (not dangerous, not useful, or not the target of attention), the brain would inhibit the input’s processing because its mental model of the stimulation needs no further updating, leading to RS (Grotheer and Kovács, 2016) (Fig. 4). The cellular mechanisms at play remain to be fully identified (Ramaswami, 2014).

To manipulate the predictions formed by the brain, researchers have developed sensory stimulation sequences with rules, *i.e.,* regularities with various levels of complexity and probability of occurrence. When adjacent (in space and/or time) inputs are identical, we speak of a local rule; if they differ, they form a local deviance. Regularities can also appear at a distal level, creating more complex patterns in the sequence called global rules. For example, a loop of four different stimuli (*e.g.*, ABCD) can be the global rule, and if a repetition (local rule) occurs suddenly in this pattern (*e.g.*, AACD), it will constitute a global deviance.

In adults, Todorovic et al. (2011) presented trials of two tones where the second tone was either identical to the first (repeated) or not and examined RS depending on whether the repetition was expected (frequent, *i.e.* the global rule in the sequence) or not (rare, *i.e.* locally deviant is the global rule in the sequence). They found a robust RS in the auditory cortices, larger when the repetition was expected than when it was unexpected. Similar results were found by Summerfield et al. (2008) using fMRI to measure RS in the visual cortex of adults presented with faces. The authors replicated this study using EEG (Summerfield et al., 2011). These results consistently demonstrate that RS is, at least partly, an active mechanism modulated by top-down information, stronger when it is consistent with the global rule of the paradigm. As an actively regulated mechanism, it is not surprising that RS interacts with attention. Larsson and Smith (2012) modified the protocol from Summerfield et al. (2008) to test the effect of attention being focused on the target stimulus *vs.* diverted from it, when the same-face condition (local repetition) was either frequent (global rule) or rare. When attention was driven onto the task the results of Summerfield et al. (2008) were replicated: RS during face repetition was robust in both conditions but stronger when repetition was the global rule. When attention was diverted away from the task, RS induced by local repetition was attenuated and there was no difference between global rules. These results suggest that attention enhances the effect of top-down information regarding global rules, and lowers the threshold of the bottom-up error signal propagation, accelerating the mental model’s adjustment from error signals. As a result of faster learning of both local and global contextual information, repetitions would become more rapidly uninformative and RS stronger with attention.

Repetition enhancement (RE) – the increase of brain activation due to the repetition of a stimulation – can coexist with RS. In the predictive coding perspective, Nordt et al. (2016) proposed that RE would appear transitorily along the course of stimulus repetition, reflecting the increased neural activity from bottom-up error signals during the formation of the mental model of the stimulus. When the model stabilizes and predictions become more accurate, error signals would become smaller, and RS would replace RE. However, multiple phenomena appear to influence the direction of the repetition effect, such as stimulus recognition or uncontrolled attention fluctuations during the task. In addition, the choice of method can have a critical impact on this phenomenon: EEG or MEG can provide either RS or RE depending on the peak considered of interest for analysis, fNIRS or fMRI can provide RS or RE depending on the temporal integration inherent to the hemodynamic response. A review of these effects can be found in (Segaert et al., 2013), showing that the mechanisms underlying the direction of the repetition effect are far from being understood.

### 3.2 The error of prediction and mismatch responses

**Fig. 5:**
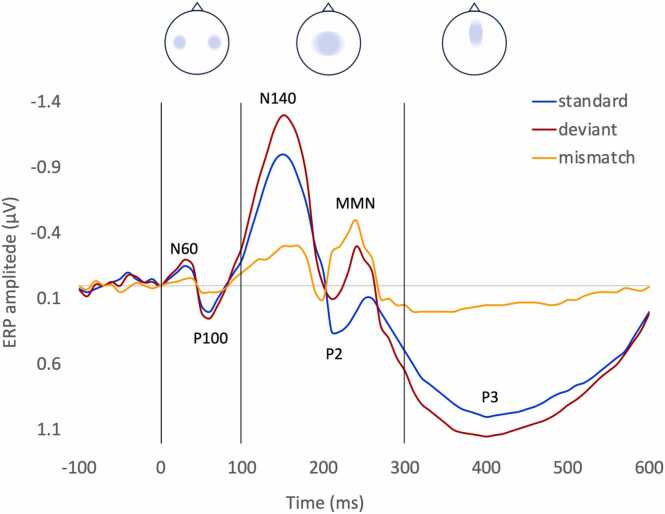
Schematic representation of a somatosensory evoked-related potential (sERP, or SEP) to a standard stimulus (blue), deviant stimulus (red), and the corresponding mismatch trace (orange). The main components are labeled. Archetypical scalp topographies of the components are illustrated above for early, intermediate, and late latencies.

In order to elicit an error signal, experimenters often use an oddball protocol in which a rare stimulus (called the “deviant”) appears randomly in a sequence of frequently repeated stimuli (called “familiar” or “standard”). The ERPs evoked by the standard and deviant stimuli are then compared in terms of components. Fig. 5 indicates components on a schematic illustration of a typical somatosensory ERP.

**Fig. 6:**
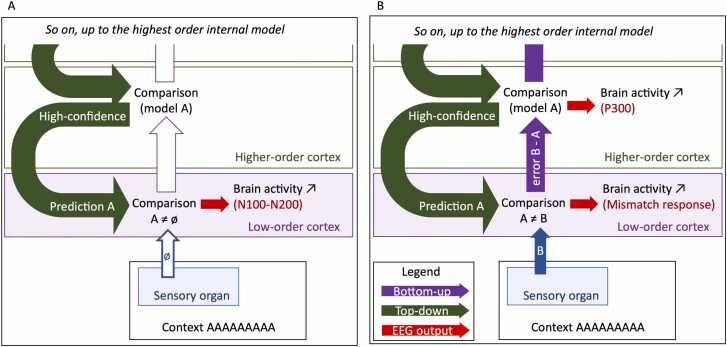
Low-order, short-latency N100 or N200 are observed when an expected stimulus is omitted. They may reflect the pure prediction signal (A). Low-order, mid-latency MMN and high-order, long-latency P300 are the result of a processed deviant stimulus: they reflect the error signal (B).

Early components, such as N60 or P100, occur within the first 100 ms after a stimulus and are typically observed in low-level (such as primary sensory) cortices. These components reflect the detection and initial processing of sensory information. As these cortices partly anticipate the input based on predictions, early components may be visible during unexpected omissions of a predicted stimulus. In omission paradigms, in which the deviation is rather an absence of the expected standard stimulus, the typical cerebral activity observed is a negative deflection 100–200ms after the onset of the omission (N1 or N2), which may reflect the pure prediction signal (Wacongne et al., 2011) or the early evaluation of the presence or absence of input (Fig. 6A).

Intermediate (or mid-latency) components, such as N140 or P2, appear approximately 100–300 ms after the stimulus onset and are associated with further analysis of the stimulus characteristics. On EEG, they are more visible on the sensory and frontocentral electrodes. Authors attempted to define the spatial location of these components by coupling EEG and fMRI (for example, Li et al., 2019; Liebenthal et al., 2003), but sources appear distributed in networks involving the association sensory cortices, the superior frontal gyrus, the anterior cingulate cortex, the insula… In a typical auditory oddball protocol in adults, a deviant tone elicits a larger negative component around 200–300ms after stimulus onset in the sensory to central electrodes. The subtraction of the average ERP to deviants from the average ERP to standards thus results in a negative peak on the mismatch trace called the mismatch negativity (MMN) (Garrido et al., 2009, Kujala et al., 2007, Näätänen et al., 2007, Stefanics et al., 2020). The deviant stimulus would violate the prediction that was based on the model built from the standard stimuli. The MMN would directly represent the error signal from low-order cortices being forwarded to higher-order cortices and be affected by local deviance (Fig. 6A).

Late components occur after 300 ms and are observed in frontal areas. The P300, commonly observed after a deviant stimulation, is a positive deflection 300 ms after stimulation that would reflect attentional engagement and/or the high-order processing of errors (Chennu et al., 2013, Riggins and Scott, 2020) (Fig. 6B). Therefore, later components like the P300 would be affected by attention and by global deviations.

### 3.3 Bottom-up vs. top-down contextual information

**Fig. 7:**
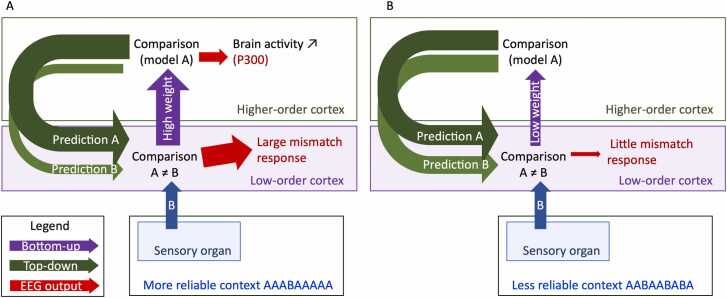
The weight attributed by low-order cortices to sensory inputs is crucial in prediction processes. In a stable, more reliable environment, a slight change of input causes a strong error signal, yielding a larger amplitude of the mismatch response (A). On the contrary, in a noisy, less reliable environment, a change of input of similar magnitude elicits a smaller, if any, brain activity, indicating that the error is not forwarded (B).

The effect of bottom-up contextual information was investigated by SanMiguel et al. (2021) who manipulated the ratio of presentation of the standard tone in a sequence. The most stable context consisted of ten identical standard tones and one deviant per block. The least stable consisted of eleven different tones, one being the target. Two moderately noisy contexts involved four and seven identical standards out of ten, with other acoustic frequencies being random. The deviant, target tone was always presented once per block of eleven tones. When the noise was high, a MMR on the P2 peak was elicited by deviants, but as standard stimuli became more frequent, the MMR to deviants became increasingly negative until it reached the characteristic MMN shape that characterizes strong error signals in adults using oddball paradigms. This suggests that the error signal is weighted depending on the current context stability, emphasizing the role of bottom-up information in low-level predictive coding processes (Fig. 7).

Top-down information also affects prediction. Todorovic et al. (2011) presented trials of two tones where the second tone was either an identical repetition of the first one or omitted. In the first condition, repetition trials occurred more frequently (75 % of the time) therefore repetition was expected (locally identical is the global rule), and in the second condition, repetition rarely occurred (25 % of the time) therefore it was unexpected. We mentioned earlier the effect of this context change on RS. In addition, they observed when repetition was expected a larger activity at 100–150ms after omission onset in the sensory cortices, compared to when the repetition was unexpected, suggesting that predictive processes in low-order cortices are also affected by top-down information conveying contextual information from higher-order cortices (such global rules, instructions, or intention), which may over-rule local predictions.

Multiple predictions can coexist based on various levels of context. Darriba et al. (2021) presented participants with sequences of tones preceded by a cue indicating the pitch of the last tone they should generate by pressing a key. If the last tone, to be actively generated, completed the ascending or descending sequence, the sensory-based prediction (local rule) was fulfilled (it was violated when an ascending tone was played in a descending sequence or inversely). If the last tone matched the cue indicating which tone to play, the intention-based prediction (global-rule from higher-order levels) was fulfilled (it was violated when the tone and cue did not match). The authors investigated brain responses to deviant stimuli when either sensory-based, intention-based, or both predictions were violated. The error signal in early ERP components was the largest when both predictions were violated, and larger when one was violated compared to when both were fulfilled. In addition, both predictions coexisted independently in the brain, as only intention-based prediction violations modulated the amplitude of the late P3b component, a sub-component of the P300, in frontal (higher-order) areas. These results suggest a hierarchy of predictions’ effects on sensory processing.

### 3.4 A hierarchy of predictions

**Fig. 8:**
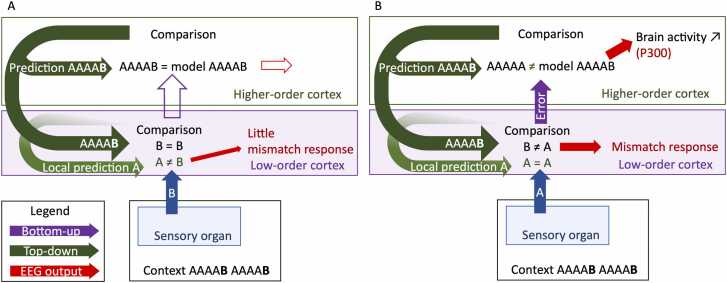
Local and global predictions co-exist and have different brain response patterns when they are violated. When only the local rule is violated, there is an increased activity in lower-order cortices (mismatch response) (A). When the global rule is violated, there is an increased activity in both low- and high-order cortices (B).

Hierarchical predictive coding means that predictions from higher-order cortices, such as those induced by global rules, instructions, or intention, may over-rule predictions from low-order cortices (local rules), such as those induced by repetition of the same sensory input (Fig. 8). A comprehensive study examined the hierarchy of brain responses in a local-global paradigm (Wacongne et al., 2011). They presented blocks of five tones either locally deviant (*e.g.*, AAAAB), locally standard (*e.g.*, AAAAA) or the fifth tone could be omitted (*e.g.*, AAAA_). During a sequence, either the locally deviant or the locally standard was presented as a global rule whereas the other block and the omission block were presented rarely. They asked participants to attend to the stimuli. They first demonstrated that the locally deviant block (AAAAB) always elicited an error signal, measured as a MMN (both in AAAAB or AAAAA global rule). The MMN was reduced when the local deviance matched the global rule (and was therefore expected). Hence, the MMN seems more sensitive to predictions from low rather than high-order levels. However, they demonstrated that locally standard blocks can also elicit a MMN, as well as a frontal P3b (a late latency peak part of the P300, when attention is caught by novelty) if locally deviant was the global rule. In the absence of a low-order prediction violation, the higher-order prediction violation can elicit error signals, suggesting that bottom-up and top-down signals are dependent on each other but also hierarchically organized. Omissions of the fifth tone revealed the prediction signals associated with this hierarchy: during omissions, the early component at 100 ms in the sensory cortex was greater when local deviance was expected. This would be due to a higher-order suppression of the predictable MMN elicited by AAAAB blocks compared with the lower-order suppression of a standard ERP. These results support a hierarchy of predictions occurring at multiple levels of sensory processing in the brain, with higher levels regulating lower levels.

It remains unclear how predictions from the high-order cortices are delivered to lower-order cortices. Friston (2018) proposed that populations of neurons involved in predictive coding communicate only with neurons at adjacent processing levels: prediction signals and error signals would be integrated at each level and the result transmitted only to the next level. In this proposition, the quick firing of neurons in sensory cortices observed when a stimulus is omitted should be preceded by increased activity in high-order cortices. However, a prior high-order prediction signal was never observed. Caucheteux et al. (2023) proposed a hierarchical model wherein predictive coding is also distributed across multiple levels in the brain, but where predictions spanning multiple timescales are formed at all levels of the prediction stream. According to the authors, simultaneous predictions would aim at different distance ranges in the brain: the nearest processing level such as in Friston’s model, for local regularities, but also long-distance predictions that would be directly transmitted across multiple levels, contextualizing short-distance predictions by providing information on global regularities. Each neuron population involved in processing would have a partial representation of the stimulus and bottom-up signaling to higher processing levels would build an integrative model of the stimulus to subsequently regulate predictions. This model of predictive coding spanning multiple timescales and ranges of neuron populations better fits the observations of hierarchical predictions in language research, but further investigations are necessary to understand the hierarchy of prediction abilities. For example, using hierarchical stimulation protocols in fMRI could inform us about the spatial hierarchy of predictive processes in different brain areas, complementing the temporal information provided by EEG.

### 3.5 Prediction and attention

Attention is a multifaceted concept designating a set of systems that regulate the neural signals’ gain, thereby regulating perception, based on the individual’s adaptive needs or goals (Narhi-Martinez et al., 2023). Attentional and predictive parameters are sometimes confounded or not distinctly reported in studies. In an attempt to overcome this, Schröger et al. (2015) proposed an operational definition for attention: *“attention is understood as a precision-weighting mechanism that regulates the gain of prediction error*, […] *attention is involved in optimizing the precision of sensory input and regulating the gain of the feedforward signal”*. They specified the role of prediction as: “*prediction is occupied with making inferences about the causes of sensory input and their expected precision”.* In other words, on the one hand, attention would increase prediction precision, possibly by quenching the variability of background neural activity in the absence of a strong bottom-up signal, as proposed by Naik et al. (2023). On the other hand, attention would increase error signal weight, facilitating the mental model's update and increasing its contribution to subsequent predictions. Thereby, prediction and attention are distinct but interdependent and interact at all levels of information processing.

Using the same hierarchical design of auditory blocks as Wacongne et al. (2011), Bekinschtein et al. (2009) used three attentional conditions: either the participants had to attend to the stimuli, or they were mind-wandering, or they were distracted by another demanding task. In the three attentional conditions, local deviance (*e.g.*, AAAAB) elicited a MMN and a P300. On the contrary, global deviance (*e.g.*, deviant block type AAAAA in frequent block type AAAAB) elicited a P300 only when attention was directed toward the task. The P300 evoked by higher-order processing of error signals seem more dependent on attention than lower-order processing of errors.

Other authors, using fMRI, argued that attention and prediction may have opposite effects on brain responses to predicted stimuli (enhancement *vs.* suppression, respectively). Kok et al., (2012) proposed a visual task in which participants had to judge gratings’ orientation. The authors orthogonally manipulated attention and prediction using two different cues before the stimulation. The first cue indicated at which side of the screen participants must look and answer during the block (attention parameter) and the second cue indicated with 75 % accuracy on which side of the screen the grating would appear (prediction parameter). They observed an interaction effect in the visual cortices: unattended predicted stimuli evoked weaker BOLD responses on fMRI than unattended unpredicted stimuli, whereas attended predicted stimuli evoked larger BOLD responses than attended unpredicted stimuli. The response to unpredicted stimuli did not change between attentional conditions: it is the response to predicted stimuli that was amplified by attention to the point of overcoming the amplitude of the response to unpredicted ones. The authors interpret their results as evidence of a synergetic effect of attention and prediction, and of attention amplifying the brain response to error signals. However, boosting error signals should not increase the response to predicted stimuli which, by definition, do not elicit an error signal. Therefore, the results of this study suggest instead that attention counteracts suppression by enhancing the processing of predicted inputs despite them not producing error signals (see also Hsu and Hämäläinen, 2021; Hsu et al., 2014, Hsu et al., 2021). It could be a mechanism underlying findings of repetition enhancement. Future studies are necessary to disentangle the possible attentional basis for repetition enhancement from other sources of enhancement relying on predictive processes or even sensitization, and to determine if the discrepancy found between studies regarding the effect of attention on low-order processing of error signals is due to imaging methods using different proxies of brain activity, stimulus presentation differences (which may also stem from the different spatial and temporal resolutions of the techniques) or analysis choices.

There is currently a limited variety of paradigms compared with the complexity of factors to be investigated, both regarding sensory modality and manipulating attention and prediction. For example, in the study by Hsu et al. (2014), two different experiments were performed which did not require the same level of attention engagement, resulting in differences in the effect on repetition suppression. We do not know if this would also happen if the prediction was cue-based (global rule) as in Kok et al. (2012). Similarly, attention can be manipulated in various ways: asking the participant to focus and/or answer to the target, to the rule, or to a distractor, may lead to different brain responses. In future experiments, authors should systematically report separately attentional and prediction factors because they do not arise from, and lead to, the same cognitive processes (Bekinschtein et al., 2009). In particular, it is important to propose different levels of prediction induction and attention engagement to be able to generalize interpretations across various situations.

### 3.6 Future directions for predictive coding research

The works we presented above raise numerous venues for research that would help us understand predictive processes in the brain and refine the model of predictive coding. Before more subtle questions are asked though, we need to harmonize the terms we use to designate concepts and their brain-measure counterparts. As we prepared this review, we were confronted with the necessity to choose one specific term among many available in the literature for every notion. This is partly due to the different epistemic origins that contributed to the field: psychology, movement science, cognitive science, clinical neuroscience… but even among developmental cognitive scientists, there is currently no consensus on terms such as repetition suppression, neural habituation, BOLD adaptation, *etc*. This makes results comparison and meta-analyses challenging.

In the process of harmonizing the terms and their definitions, we may reveal inconsistencies in how the mechanisms are conceptualized between studies that use different stimulation protocols or different brain activity measures. An important step in unraveling predictive coding will be to precisely identify the correspondence between its various constituents – low-order *vs.* high-order error processing, local *vs.* global rule prediction, attention-specific signals – and how they translate into ERPs (EEG and MEG) and hemodynamic changes (fMRI and fNIRS). This requires elaborating stimulation paradigms involving multiple levels of deviance (from a hierarchy of local *vs.* global regularities), degrees of input reliability (bottom-up contextual information), implicit *vs.* explicit cueing conditions (top-down contextual information), and exogenous *vs.* endogenous control of attention… in every sensory modality.

In progressing beyond the oddball, we also need to increase the complexity of the mental models to be formed, using complex items and categories as in Grossmann et al. (2009) for example, and more ecological context information such as real-life goals for top-down information, and realistic environmental noise for bottom-up information. More ecological paradigms will also involve intermodal mental models, which are necessary to study how predictions in multiple sensory modalities interact and contribute to an integrative sensory perception of real-life environments. Along the way towards ecological paradigms, introducing action control will again challenge the terms and concepts of predictive coding as we need to accommodate the efference copy, forward model, or agency concepts. An important methodological step will then be to consider the role of the cerebellum in both movement and cognitive control (Sokolov et al., 2017, Welniarz et al., 2021). Is the cerebellum a part of the distributed prediction stream and if so, at which order? Purely sensory experiments tend to only assess sensory cortices and the frontal areas where the MMR and P300 components are mostly seen, but there may be other valuable signals, for example in the frequency domain, in other areas that would inform us on predictive mechanisms with a finer grain. This may also allow us to determine whether prediction takes place from near to near as proposed by Friston (2005) or in parallel at multiple distances as proposed by Caucheteux et al. (2023).

Mapping the correspondence between mechanisms and functional measures, as well as understanding the processing steps in detail both temporally and spatially in the brain, could be achieved by multimodal imaging studies, of which there are still too few. Thanks to its high temporal resolution, EEG allows single-trial analysis with short intervals and the analysis of the successive phases of sensory processing. However, its spatial resolution is limited, and it is difficult to distinguish parallel sources of neural activity especially if they are spatially close. On the other hand, fNIRS and fMRI rely on the hemodynamic/BOLD response, which integrates all neural activity even without synchronous firing, acting as a high-pass filter. Its temporal resolution is low but fMRI has a good spatial resolution, can localize processes and could inform on deep and cerebellar mechanisms, whereas fNIRS allows for more ecological experimental settings. In addition, both are more appropriate for complex or long stimuli. It would be tremendously helpful to combine EEG with fMRI, for example to distinguish error signals’ localization according to the hierarchical level of the deviance (local *vs.* global). We could also analyze temporal and spatial variations in repetition effects (RS vs. RE) that do not correspond to the same underlying neural changes when measured with ERP or BOLD amplitudes. Likely, RE of the BOLD signal does not correspond to RE in all components of ERPs. Simultaneous EEG-fMRI remains a challenge, but suitable EEG signal acquisition and processing tools are being developed (Bullock et al., 2021).

Finally, recent work shows that systemic (cardiac and respiratory) rhythms influence sensory processing as there are interactions between interoceptive and exteroceptive processes that modify brain responses to stimuli depending on the phase of systemic variables at stimulus onset (Grund et al., 2022). These interactions probably influence predictive processing, but an embodied approach to predictive coding has yet to be explored.

## 4 Predictive coding in child development

As we begin to unravel prediction processes and how they interact with attention in adults, we are still far from understanding how they emerge and interact during child development, but there is increasing consensus that they would be the mechanisms by which infants actively engage in sensory processing of their environment (Baek et al., 2020). Whereas RS has been described from the youngest age, other aspects of prediction processes in development have yet to be revealed.

### 4.1 Habituation and repetition suppression in early child development

Habituation is a behavioral response decrement of any kind that results from repeated stimulation (Rankin et al., 2009). It was proposed as the simplest and most common form of learning, useful to save cognitive and physiological resources (Bubic et al., 2010). Behavioral habituation was first used to show discrimination abilities in infants: after a familiarization phase when a stimulus is repeatedly presented until the habituation criterium is reached, the test phase consists of the simultaneous presentation of two stimuli, the familiar one and a novel one. A longer exploration time of the new stimulus, called dishabituation, is interpreted as novelty preference – children would seize the novelty as an opportunity to learn – and shows the child’s ability to distinguish them. Using this approach, research on infant cognition and perception has uncovered a wide variety of abilities and knowledge about the world from an early age (Sicard-Cras et al., 2022). Indeed, a hyperbolic learning pattern is observable in two-day-old newborns: they turned their heads more frequently toward a repeated sound during the first trials but rapidly stopped. The orienting response returned only when a discrepant sound frequency was presented (Weiss et al., 1988). The more discrepant the sound from the familiar stimulus, the more infants oriented, until the proportion of trials with a head turn reached an asymptote. Authors proposed that highly deviant stimuli would require excessive cognitive resources, therefore becoming aversive rather than attractive. Novelty preference leading to dishabituation has been extensively used to study early development, such as infant tactile processing (Dumont et al., 2017), visual processing (for a review see Kavšek and Bornstein, 2010), language processing (for a review see Gervain and Mehler, 2010), and even category processing (Grossmann et al., 2009).

RS has been presented as the neural analog to behavioral habituation (Hartkopf et al., 2019, Nordt et al., 2016). RS appears similar to adults in children from three years of age (Espenhahn et al., 2021). Auditory RS is already described in fetuses using magnetoencephalography (MEG) with simple tones from 30 weeks of gestation (Muenssinger et al., 2013) and in six-month-old infants using fNIRS linguistic stimuli (Emberson et al., 2019). Similarities were observed in experimental results when using RS or habituation. For example, when the familiarization phase is too short for a stable representation to be formed (incomplete learning), infants tend to keep looking at the familiar stimulus rather than the novel one (Nordt et al., 2016). This familiarity preference would be the behavioral equivalent of repetition enhancement, when the mental model is still being formed. Grossmann et al. (2009) tested both habituation and RS in six-month-old infants using categories that require high-order level processing. During the familiarization phase, they presented various images from a category (*e.g.,* birds). During the test phase, infants were presented with items they had seen, new items from the same category, or items from a new category (*e.g.,* fishes). The behavioral analysis revealed novelty preference at the category level: images from the new category resulted in a longer looking time than new images from the familiar category. The neural responses analysis showed that, during the familiarization phase, repeated items elicited RE of the early positive component in the visual cortex, whereas during the test phase, images they had seen elicited a RS of this early component, supporting the predictive coding view at low-order levels of RE reflecting increased neural activity from bottom-up error signals during the formation of the mental model of the stimulus, to be replaced by RS after the model’s stabilization (Nordt et al., 2016).

### 4.2 Sensory prediction experiments in typically developing children

Restuccia et al. (2009) described responses to deviance and omission in children from six to eleven years of age using a somatosensory stimulus (electrical impulse on the fingers, deviance consisted of a change of the stimulated finger) while attention was engaged in another demanding task. ERPs revealed similar signals to those described in adults: deviance elicited a MMN associated with a negative frontal activity 220 ms post-stimulation, reflecting error signals, and omission elicited a N140 reflecting the prediction signal in somatosensory areas. Using an auditory protocol, Gonzalez-Gadea et al. (2015) presented children and adolescents from eight to 15 years of age with three types of sequences: standard sequences presented in one ear (*i.e.,* AAAAA), expected deviants presented in the same ear as standards (*i.e.,* AAAA**B**), that subjects had to attend to and count, and unexpected deviants presented in the opposite ear (*i.e.,* AAAA*B*). The expected deviants elicited a MMN, and both types of deviants produced a P300 but of greater amplitude for expected deviants, attention seemingly playing the same role as it does in adults of facilitating the propagation of error signals. However, prediction and attention cannot be fully disentangled in this paradigm.

Dercksen et al. (2022) compared children from six to eight years of age and adults using an auditory protocol where subjects pressed the button that triggered a sound. This type of design interestingly bridges the predictive coding and the action control types of paradigms, with sensory inputs resulting from voluntary movements. The temporal component of the prediction thus relies on the subject’s intentions (top-down information) rather than the temporal regularity of the sequence (bottom-up information), while only the type of sound stimulus is manipulated. When the button was pressed, a single sound could be repeated (single sound condition) or random sounds were presented each time (random sound condition). The sound was unexpectedly omitted 15 % of the time. Children and adults displayed the same pattern during the sound omission in the auditory cortex, a negative activity 90–200ms after omission onset, although the responses were slower and wider in children. Furthermore, brain responses differed between the single and random sound conditions, with the response to omission being larger for the single sound condition. This was discussed as the result of a precision-weighting mechanism that would calibrate prediction signals depending on confidence: in a reliable environment, predictions are more reliable and therefore given more weight in input processing. As a result, a larger prediction signal is visible during omissions, in children as in adults.

We could not find any experimental study of sensory prediction in typical children between two and six years old. This age gap needs investigation to outline the developmental trajectory of these abilities and understand how they relate to attention and executive functions, which both develop most rapidly during this period (Fiske and Holmboe, 2019, Rueda et al., 2004).

### 4.3 Sensory prediction processes in infants

Infants’ predictive abilities have been investigated more extensively than school-age children’s. In six-month-old infants, Nelson et al. (1990) used sequences in which a visual stimulus frequently followed an auditory cue. The picture was omitted 20 % of the time. No prediction signal was seen on EEG during the omissions but there was an enhanced activity at short and late latencies on the post-omission trial (the standard trial immediately following the omission), indicating that the omission had been processed. With a similar bimodal oddball protocol in six-month-old infants, but using fNIRS, Emberson et al. (2015) observed increased oxygenated hemoglobin in the visual cortex after an auditory cue, during unexpected omissions of the visual stimulus previously associated with the cue. This suggests that top-down information from a mental model of the bimodal association already modulates sensory prediction at six months of age.

Furthermore, Basirat et al. (2014) found that three-month-old babies were able to form hierarchical predictions in a unimodal paradigm. They used blocks of four tones with either a locally deviant (*e.g.,* AAAB) or locally standard (*e.g.,* AAAA) final tone, that were presented with a ratio of 75–25 %, counterbalanced. Therefore, there were also global-rule deviances *(e.g.,* deviant AAAA among AAAB frequent sequences). Results showed a MMR for both types of deviance, stronger for the rare local deviance (when both local and global rules are violated), supporting the MMR reflecting additive error signals. In addition, a late negative bilateral activation in the anterior frontal cortex was specific to global-rule deviance (locally standard sequence when locally deviant was the global rule). These results show a hierarchy of predictive processes comparable to that of adults as early as three months of age, only with longer latencies.

Dehaene-Lambertz and Dehaene (1994) used an oddball auditory protocol in two-to-three-month-old babies. The sequences of five stimuli were composed of four identical phonemes and a fifth that was either were the same (/ba) or locally deviant (/ga). During the homogenous sequences, a significant RS across repetitions was observed at both short and long latencies whereas during the deviant stimulus, there was an enhanced amplitude of both peaks and a late frontal activity. This indicates an error signal in the auditory domain using an oddball paradigm. The authors concluded from the late frontal activity that two-to-three-month-old babies could already have attention-orienting processes analogous to the P300. Similar results were found in one-year-old infants using a cross-modal association of a sound predicting an image with 75 % accuracy (Kouider et al., 2015). Infants exhibited a greater early P1 component to cued images compared with unexpected ones, suggesting the cue acted as an attention arousal trigger, whereas they presented a late slow wave when images were unpredicted. These results were obtained with different types of stimuli (phonemes *vs.* faces, respectively) at different ages (3 *vs.* 12 months), and thus yielded different ERP shapes where peaks are not directly comparable, limiting the interpretation of their similarities, but they both suggest that infants’ predictive processes may be sensitive to attention allocation even when attention is exogenously rather than endogenously controlled. In the study of Grossmann et al. (2009) testing prediction in categories instead of single stimuli (see *habituation and RS* section above), new items of the familiar category during the test evoked an enhanced late component only, whereas images from a new category elicited a negative component (Nc) at intermediate-latency on central electrodes and a late positive component (LPC) at late latencies, which may be considered immature equivalents of the MMR and the P300 (Riggins and Scott, 2020), also supporting the early development of high-order predictive or attentional sensory processing. However, we must stay cautious with this interpretation as the correspondence between infant ERP peaks and the adult P300 remains open to debate, both in terms of the corresponding cognitive function and of the underlying neural substrates.

### 4.4 Sensory prediction and cognitive development

In a meta-analysis on visual habituation/dishabituation, Kavšek (2004) indicated that performances in such paradigms during infancy could be predictive of intellectual quotient at preschool or school age. Precisely, habituation performance would be a better predictor in low-risk infants whereas dishabituation/novelty preference performances would better predict intelligence in high-risk infants (due to premature birth, low birth weight or failure to thrive). Poli et al. (2023) showed that eight-month-old infants with high processing speed needed less time to habituate to repeated stimuli, corroborating the association between habituation and intelligence found by Kavšek (2004). Further, they showed that infants who did not habituate but were curious (defined as a bias towards information-rich stimuli, operationalized as the correlation coefficient between the information content of a stimulus and the subject’s looking time) still showed a novelty preference rather than a familiarity preference. Curiosity could have a preventive effect, compensating for the lack of habituation. To assess whether habituation and dishabituation are predictors of cognitive development, longitudinal studies are needed, particularly during early development in babies prone to neurodevelopmental disorders.

These behavioral studies would benefit from a neuroimaging approach to refine our understanding of the link between prediction and cognition. Wang et al. (2022) used an auditory-visual oddball paradigm in which an image usually following a sound is sometimes omitted, and recorded cortical hemodynamic activity using fNIRS in the visual cortex of six-month-old babies (similar protocol to Emberson et al., 2015). They observed a correlation between the amplitude of the brain activation during omissions and language development at 12 and 18 months. In neonates, Schwarzlose et al. (2023) using fMRI and an auditory oddball protocol reported that larger BOLD responses to deviants were related to behavioral inhibition, a precursor of anxiety, at 1 year of age. These findings are exciting as they open new venues of research to understand neurodevelopment and its behavioral and clinical manifestations. Unfortunately, the temporal resolution of the hemodynamic measures does not allow to interpret this result in terms of the prediction signal (short latencies on ERPs) or error signal (intermediate and late latencies on ERPs) being associated with language development. More studies with varied stimulation protocols and other imaging methods are necessary to explore the role of prediction in cognitive development.

### 4.5 Interactions between prediction and attention during early development

Attention shows a protracted development. Posner’s model of attention draws a parallel between behavioral observations and brain networks considered the substrates of attention, and subsequent work showed that these networks have different developmental trajectories that parallel attention development in childhood (Rueda et al., 2004). The first to develop is the salience network, the substrate of alerting attention, which regulates behavioral responses to any stimulus, and orienting attention, which regulates behavioral responses to stimuli that are more likely to be relevant from an adaptive standpoint (Schimmelpfennig et al., 2023). During the first months and years of life, infants learn from experience to select relevant information in their environment, starting with the control of eye gaze to actively focus their attention and explore targeted objects (Colombo, 2001). In other words, attention initially depends on stimulus salience, relying on bottom-up information, then becomes increasingly ruled by high-order cortices at the service of intentions. The increased endogenous control of orienting is a fundamental prerequisite for the development of executive attention, which relies on a different network involving the prefrontal cortex, and sustains the rapid development of executive functions such as inhibition or switching (Posner and Rothbart, 2014). These functions are core cognitive processes critical for learning complex skills, school achievement, social interactions (Diamond, 2013) and autoregulation strategies (Rothbart et al., 2011). It is therefore not surprising that sustained attention performance at one year of age is a predictor of effortful control at two years (Johansson et al., 2015).

The interactions between prediction and attention in early child development are still speculative and remain to be investigated. The difficulty resides mostly in measuring attentional processes early in life, and in the paucity of paradigms appropriate to young participants in terms of duration and content. The dramatic transformations in the way attention is controlled, and expressed behaviorally in the early years of life, implies that studying attention and its interactions with prediction requires different paradigms at different ages. Attempts at measuring attention in preschoolers have been made (Mahone and Schneider, 2012, Weiss et al., 2018) but under three years of age, quantification is elusive. This is partly due to the lack of consensus on what attention is (Wu, 2023) and partly because during development the networks may not be distinct, in particular orienting and executive attention may be overlapping in the first years (Petersen and Posner, 2012). However, there is recent evidence of precursors of sustained attention during the first months of life (Holmboe et al., 2018), suggesting it is developing much earlier than what we are currently able to quantify. Given the importance of attention in early and late development, and evidence of prediction as early as a few months of age, we can assume that attention and prediction develop jointly: prediction may facilitate the emergence of attention-orienting processes by learning environmental patterns and allowing the arousal of attention by unexpected events, and attention may facilitate the development of prediction abilities by optimizing sensory inputs, especially in volatile environments. Unfortunately, very few studies are available, and the two notions are often entangled either at the conceptual or at the experimental level, or when interpreting the results. For example, in Martinez-Alvarez et al. (2017) the sentence *“In terms of the underlying mechanisms, the main characteristic of endogenous attention is that it is oriented based on internal expectations.”* implies that prediction (referred to as “expectation”) would be a mechanism of attention. Then, the authors go on to write *“These expectations can be driven by the stimulus presentation itself (stimulus-driven expectations) or can be driven by internal predictions based on previous experience/learning (goal-directed or experience-based expectations)”.* In that sentence, *stimulus-driven expectation* is akin to local prediction, which also depends on experience and learning. Expectation does not spontaneously arise from the first stimulus ever encountered by the brain but after the repetition of similar sensory events. The authors use *experience-based expectations* to refer to higher-order predictions, but the dichotomy does not reflect that learning and experience occur at all levels of the predictive process, and guide attention but are not its mechanism. Nonetheless, the authors showed that 12-month-old infants were able to anticipate where, but not when, a stimulus appeared following a symbolic cue with both spatial and temporal cueing value, whereas 15-month-olds anticipated both location and temporal information. This very interesting result suggests that temporal prediction may have a protracted development compared with spatial prediction in the visual domain. In the article, however, it is only discussed in terms of attention orienting, not in terms of sensory prediction mechanisms. In studies using hemodynamic measures, the “neural response to novelty” in a unimodal oddball paradigm (Schwarzlose et al., 2023) or the “cortex response to unexpected omission” in a bimodal oddball paradigm (Emberson et al., 2015) integrate both the predictive signals and the attentional effects in the measure. Further studies examining the interaction of prediction and attention in typically developing children and infants are needed to understand how these skills contribute to cognitive development.

### 4.6 Future directions for predictive coding in child development

First, there is a need to disentangle the effects of attentional processes from predictive processes by orthogonally manipulating attention in typically and atypically developing children and reporting how attention was manipulated as it can be affected by the reliability of bottom-up contextual information, and in turn, affect the prediction precision and the error signal. We must consider attention components of experimental paradigms and results separately from other top-down information, such as high-order predictions based on global rules or cues. To date, we could not find any study that purposely examined the interaction of attention and prediction in children. We could not find any in-depth study of sensory prediction in children between two and six years old either. This age gap needs investigation to outline the developmental trajectory of prediction abilities, and a priority at that age will be to understand how they relate to executive attention and functions, which develop most rapidly during this period and are the crucial foundation of school achievement (Fiske and Holmboe, 2019, Rueda et al., 2004).

Designing ways of controlling attention engagement in toddlers is no little endeavor because exogenous attention through orienting to salient stimuli still exerts a strong influence during that developmental period: explicit instructions cannot be effective, contrary to older subjects. To distract attention away from the input of interest, using salient visual stimuli such as cartoons while testing in the auditory or somatosensory modalities can work when using fMRI, engaging in play is a good alternative when using EEG or fNIRS. To attract attention towards the input of interest, new multisensory ecological stimulation protocols are needed. In the field of executive function development, researchers have recently proposed promising tools using playful interactive content on electronic devices for toddlers (Fiske et al., 2022). Similar tools could be designed to manipulate and measure prediction and attention. It will also be useful to take advantage of eye-tracking techniques to control for temporary loss of attention.

Studying toddlers and older children can also help us understand the developmental trajectories of the various components of predictive coding. For example, if RE does indeed reflect the transitional period of mental model formation, it will likely appear stronger at different ages depending on stimulus complexity (at the trial level, for example categories of animals *vs.* colored dots) and rule hierarchy (at the sequence level, for example more subtle regularities to extract). Another example is the P300 ERP component that is commonly associated with attention arousal in older children and adults, for which the infant literature does not provide a consensual equivalent (Riggins and Scott, 2020), and we found no reference at preschool age with predictive paradigms. The developmental evolution of the brain correlates of sensory prediction is still a mostly untapped resource to uncover foundations of neurodevelopment, and this is also unfortunate because it means we lack normative data with which to compare the data of children with ND.

## 5 Implications for neurodevelopmental disorders

### 5.1 Predictive coding as a theoretical explanation for autism spectrum disorders

The real world is quite volatile and requires that the individual quickly adjusts to change, yet the complexity of the physical and social environments does not allow for purely reactive behavior, which would be inefficient in the long term. Some learning (improving performance while reducing flexibility) is necessary to optimize behavior based on *a priori* estimation of events likelihood. High-functioning prediction abilities derive from building this compromise between efficacy and plasticity. Impairment in finding the optimal compromise can make all life experiences stressful and even overwhelming.

Ten years ago, several authors proposed that NDs’ origin and symptoms, especially those found in ASD, could be explained using the predictive coding framework. Pellicano and Burr (2012) proposed that individuals with ASD would use less *a priori* constraints (“hypo-priors”) when analyzing sensory information and therefore their perception would rely more heavily on real inputs (bottom-up information) than on predictions, compared with typical individuals. As a result, their perception would be less biased by top-down information, but strenuous. This would explain exceptional performance in sensory detection tasks but sensory overload in real-life situations. Sinha et al. (2014) proposed that ASD symptoms may result from a failure to learn transitional probabilities in the environment and generalize them to new situations, making future predictions impossible. Without being able to take prior knowledge into account to habituate, the environment would always be unpredictable and stressful, which could explain many ASD symptoms. Stimming behaviors (stereotyped and repetitive gestures such as hand flapping or spinning) can particularly be explained using this framework. Stimming could be a way to facilitate prediction by decreasing sensory volatility to a minimum. Insistence on sameness would also serve this function. Unpredictability could also explain sensory hypersensitivities (Zoenen and Delvenne, 2018). Reduced stimulus predictability, which derives from impaired prediction and habituation, would make social touch or loud noises overwhelming and stressful (Sinha et al., 2014). Cascio et al. (2015) tested tactile sensitivity in children aged from 5 to 17, with or without ASD. They showed that hypo- and hyper- tactile sensitivity, as well as ASD symptom severity, were linked to the latency of ERPs to a puff of air. In the ASD group only, tactile hypo-responsiveness was associated with a reduced late component of the response, which could reflect impairment in higher-order (late sensory and/or early attentional) processes, consistent with reduced perception. On the contrary, hyper-responsiveness was associated with shorter latencies of the early components, suggesting faster low-order processes, although this association was not significant. Despite converging lines of research, it is still unclear whether prediction plays a direct role in sensory reactivity in ASD. Recent attempts to investigate this specific question in adult patients with low-severity ASD have yielded disappointing results. For example, Cannon et al. (2023) tested if ASD adults benefited less than controls from cues or rhythmic presentation in an auditory detection task but found that the “predictive boost” was attenuated only when patients had concurrent ADHD.

Van de Cruys et al. (2014) and Lawson et al. (2014) situated the core deficit of ASD not in the ability to build a mental model or generate predictions from explicit rules, but in a high, inflexible weight assigned to error signals, that would hinder high-order learning and generalization in naturalistic settings where most rules are implicit. Because all error signals (bottom-up information) would be given the same high weight, patients would not be able to differentiate informative errors that make the world learnable and later predictable, from the noise that is unlikely to be applicable in the future. Lawson et al. (2017) provided experimental support to this idea. In their study, ASD individuals made fewer mistakes with unexpected targets than control peers during a probabilistic associative learning task. The authors discussed this result as an overestimate of the noise by people with ASD, who relied less than controls on bottom-up information to infer the underlying rule and build a model of the associations, leading to the inability to distinguish surprising deviants from overall volatility. This also explains that they would perform better than normal at noticing all low-level (local) changes while having trouble establishing higher-level (global) rules when such rules are implicit, to be extracted from noisy bottom-up information. Goris et al. (2018) used a local-global paradigm like the ones of Chennu et al. (2013) or Basirat et al. (2014) and showed that in adults with ASD, the global rule exerted a weaker modulation of the MMN induced by local deviance, compared with controls, suggesting a difficulty to assign a context-dependent weight to the local error signals. However, the results are inconsistent. Goris et al. (2018) found lower MMN in general in adults with ASD, supporting the view of equal weighting of bottom-up information, both sensory inputs and error signals, but no difference with controls on the P3b response, suggesting that high-order processes may be preserved. Gonzalez-Gadea et al. (2015) had previously found no difference in MMN between ASD, ADHD and typical teenagers, but a lower P300 to unexpected deviants and higher P300 to expected deviants in ASD compared with other groups, which is consistent with higher weight of all bottom-up information but an impaired ability to use implicit rules for faster subsequent processing of standard stimuli. Because participants were not the same age in these studies (adults *vs.* 8–15 years), developmental effects may be at work that would explain why the same pathological mechanism manifests itself at different levels of the processing stream. Further studies on top-down processes are needed to disentangle paradigm, ND and age effects on the MMN and P300.

Van de Cruys et al. (2014) introduced attention as a mechanism to understand the atypical weighing of error signals. ASD people often show increased performance in selective attention, which could make deviant stimuli overly salient and reinforce high-weight attribution to error signals. However, this advantage would be counter-productive in a noisy, less predictable, naturalistic environment, that requires a more flexible allocation of attention. Todorova et al. (2021) used an apparent motion paradigm to create the illusion of moving objects that could be predictable or unpredictable. Participants with ASD were asked to press a button every time a target appeared regardless of its predictability. Descriptive analysis showed that most of the autistic individuals were able to form predictions, but they had a faster reaction time than controls to the unpredictable targets. These results are supported by another study using a visual-auditory omission paradigm in adults with ASD (van Laarhoven et al., 2020). Results demonstrated that they had larger early ERP components in the sensory area in response to the unexpected omission of the sound during the presentation of a handclap, compared to controls. Taken together, these studies suggest that people with ASD can form predictions, but that they are tuned to local changes, maybe due to atypical attention, so that their responses to unpredictable events differ from those of typical people. Indeed, people with ASD can have attentional impairments: they engage with irrelevant stimuli and have trouble disengaging from them (Elsabbagh et al., 2009). The authors also proposed that social impairment, the core symptom in ASD, could also be understood through this theory. Social interactions are highly complex, encompassing several processes in parallel notably emotion processing, speech processing, body language processing, and theory of mind. In addition, these inputs can contradict each other, like in humor, and most social rules, including irony, are implicit. This requires highly flexible attention allocation, and also the ability to extract regularities from bottom-up information whereas many inputs in social situations are uninformative and should be lower weighted in order to extract relevant cues.

The proposals are not mutually exclusive: a systematically high error signal weight, as in the proposal by Van de Cruys et al. (2014), would prevent model stabilization, leading to systematically low prediction precision as in the proposal by Sinha et al. (2014), and poorly-guided perception as proposed by Pellicano and Burr (2012). A recent review of experimental attempts at testing these theories can be found in Shi et al. (2024). The authors report some agreement among studies on patients with ASD having deficits in extracting global rules, however, top-down information from explicit cues or knowledge does not seem affected. The results of their experiment support the inflexible error signal weighting proposed by Van de Cruys et al. (2014).

Although we are still far from experimental validation, these various angles of explanation provide avenues for neurodevelopmental investigations in populations at risk, before the age of diagnosis. Indeed, sensory prediction can be evaluated earlier than behavioral symptoms. For example, Piccardi et al. (2021) examined neuronal responses to a vibrotactile RS protocol in ten-month-old infants who had elevated *vs.* typical likelihood of ASD or ADHD (based on having a first-degree relative diagnosed with either). Results showed that infants with an elevated likelihood of ASD had reduced RS compared to the other groups, and that was predictive of ASD traits at 24 months, supporting the hypothesis that they have less stable mental models from a young age. Indeed, in adults, Ewbank et al. (2015) also found a reduced RS for visual stimuli such as faces, scenes, and even geometrical objects as a function of autistic traits. Interestingly, this effect was found despite changes in stimuli display size, suggesting the effect cannot entirely be attributed to the low-level properties of the stimuli but may occur at all levels of the processing stream. It will be necessary to further assess impairment in the different steps of predictive coding processing of sensory stimuli, at various ages and levels of the pathological process, to precisely understand the causal order of impairments leading to ASD symptoms.

### 5.2 Predictive coding and ADHD

No strong theoretical proposition has been made regarding the role of prediction in other ND. Following those we presented for ASD, we can however infer predictive coding impairments in ADHD. ADHD’s main symptoms are difficulties in controlling attentional focus and inhibiting distractors (Crocq and Guelfi, 2015). In the predictive coding framework, it would be associated with a weaker P300 response during deviance compared to typical individuals, a result that was indeed observed in several experiments (Peisch et al., 2021, Senderecka et al., 2012, Stevens et al., 2007). ADHD symptoms could be explained by a low precision attributed to the prediction formed from the standard stimulations, and a high weight attributed to novel bottom-up information, resulting in greater responses to unexpected stimuli. Gonzalez-Gadea et al. (2015) found that opposite to ASD, ADHD was associated with decreased P300 but increased late frontal activation to unexpected stimuli, associated with higher set-shifting skills. The authors proposed that the underestimation of prediction precision and over-weighting of error signals could be associated with a pathological failure to control task switching, enhancing distractibility. To date, there are very few studies of these phenomena and their results are not fully consistent, therefore we must stay cautious with these interpretations. With the development of research on predictive coding in children and infants, we expect that future studies will be able to describe early pathological sensory processing that may link prediction impairments with the emergence of clinical symptoms. It will be crucial then to clarify which steps of the predictive process are specifically impaired in each syndrome, and also to identify the common alterations that could explain the frequent comorbidities (Taurines et al., 2012). If both ADHD and ASD are indeed characterized by a high weight of error signals but differ in other aspects, this would help target early interventions in children who are susceptible to any ND, such as premature neonates (Johnson and Marlow, 2011). The authors who investigated predictive coding as a core deficit in ND highlight the potential of this line of research to uncover very early cross-syndromic markers of ND (Gargaro et al., 2011, Gonzalez-Gadea et al., 2015, Piccardi et al., 2021, Taurines et al., 2012) and recent work supports the transdiagnostic nature of sensory processing profiles, showing similar clustering between ASD and ADHD (Scheerer et al., 2024). In the future, it will also be necessary to investigate prediction alterations in other ND such as specific learning disorders, neurodevelopmental motor disorders, or the dysexecutive syndrome of prematurely born children, to get a broader understanding of early predictive impairments in neurodevelopmental disorders and how they can be used for early diagnosis and intervention. With these goals in mind, authors have focused their efforts on characterizing predictive coding in the neonatal period.

### 5.3 Prediction as an early screening marker

We have seen that toddlers and infants are able to form predictions. A detailed account of the many behavioral paradigms of infant cognitive research that can be interpreted in terms of prediction (statistical learning, associative learning, operant conditioning, “impossible event” paradigms, *etc.*) can be found in Köster et al. (2020). Research using measures of brain activity described predictive mechanisms in neonates. Háden et al. (2012) reported an activity at central electrodes in newborns during rare omissions of a tone in a repetitive sequence of drums, and Háden et al. (2015) showed that a rare discrepant ascending pitch in a descending train of tones elicited a mismatch response. These studies support the idea that from birth, non-noxious stimuli are processed in a predictive manner, and that discrepant ones (new, oddball or missing) elicit an error signal. Manipulating bottom-up information, Winkler et al. (2003) showed that, like adults, newborns need a stable sensory environment to extract patterns and display a mismatch response to deviant tones. Winkler et al. (2009) showed that the prediction signal was observed during sound omissions only when the missing stimuli were perceptually salient (on the beat). In the same way as adults, newborns might also weigh error signals, the weight increasing with context stability, giving them the ability to anticipate a musical beat.

The challenge now is to dig deeper into the emergence of predictive processes in newborns vulnerable to ND, such as premature neonates but also siblings of diagnosed children, children at risk due to socio-economic status or trauma exposure, and children with chromosomal anomalies. We should follow them up into toddlerhood when ND symptoms manifest to understand how early prediction deficits can alter neurodevelopment. In such populations, a priority is to identify sensitive periods of sensory processing that are particularly vulnerable to deleterious sensory inputs such as painful care procedures, abuse, or toxic exposure, and evaluate how these risk factors specifically affect predictive coding development. Several factors may be responsible for an accelerated closure of sensory processing sensitive periods such as early exposure to visual stimuli in premature neonates (Mowery et al., 2016) or prenatal exposure to selective serotonin reuptake inhibitors (Weikum et al., 2012). The consequences of modified sensory cortices maturation on predictive coding and the subsequent development of multimodal processing regulation, attention and cognition, remain unknown.

### 5.4 Predictive coding in premature infants

Premature birth is defined as a birth occurring before 37 weeks of gestational age (GA) and represents about 10 % of births worldwide. Prematurity is associated with an increased risk of psychiatric disorders, especially ND. Following up a cohort of extremely premature children, Johnson and Marlow (2011) found that at 11 years old, children born extremely premature (before 27 weeks GA) had at least a threefold increased risk of developing any kind of psychiatric disorder including ND and particularly ASD and ADHD. Prematurity is also associated with poorer global development, language delays from multiple risk factors associated with prematurity (Coughlan et al., 2023, Spek et al., 2012), lower school achievement (Allotey et al., 2018). Children born prematurely, like children with ND, frequently develop atypical sensory processing. Multiple studies reported particularly auditory and tactile impairments (de Paula Machado et al., 2019, Rahkonen et al., 2015). Not only did preschoolers who were born preterm have more frequent atypical parent-reported sensory profile scores, but these were associated with atypical scores in executive functions such as working memory or inhibition (Adams et al., 2015), functions that are also impaired in ND. Overlapping sensory deficits in children with ND and children born prematurely suggest shared sensory mechanisms. Sensory prediction deficit may be such a mechanism.

Emberson et al. (2017) studied sensory prediction using fNIRS in six-month-old infants born at term or preterm using their auditory-visual omission protocol. In infants born at full term, they observed an increase in oxygenated hemoglobin concentration in the visual cortex during the omission of the expected visual stimulus, similar to the response to presented stimuli, which the authors interpreted as a prediction signal. In infants born preterm there was a small decrease instead, suggesting that preterm infants did not provide prediction signals to the visual cortex, although a behavioral control experiment using looking times showed they were able to learn the bimodal association In their subsequent study using a comparable protocol, Boldin et al. (2018) examined the hemodynamic changes occurring when the infants were learning the cross-modal association. They reported that babies from five to seven months of age had different learning trajectories in the occipital cortex depending on neurodevelopmental status: term-born infants had an inverted U-shape for the neurovascular response amplitude across learning blocks, whereas preterm-born infants had a horizontal neural learning trajectory, only in this cortex. This flat learning curve predicted the lower occipital response to unexpected omissions, supporting an impaired ability in preterm infants to build a mental model for generating *a priori* visual predictions based on the auditory cue, during repeated presentations, despite being able to recognize the association *a posteriori* in the behavioral condition. The authors emphasize that early deficits in sensory prediction and learning may be a reason why preterm infants are at elevated risk for altered neurodevelopmental trajectories.

Although these studies suggest the effects of premature birth on prediction, they were conducted on infants several months old who were born preterm, not neonates. Edalati et al. (2022) used EEG to test neonates born between 30 and 33 weeks GA a few days after birth, around 33 weeks corrected GA, during an auditory oddball protocol where the trials were deviant because of their rhythmic presentation (79 % of trials were presented with standard 1000–500–500 ms inter-tones intervals, whereas 21 % of trials were presented with deviant 1000–250–750 ms intervals). Results showed a positive MMR in frontocentral electrodes. A subsequent negative deflection 400–500ms after deviants was followed by a late positive component (LPC) on frontocentral electrodes, which could relate to the P300 activity found in children and adults (Riggins and Scott, 2020), suggesting that high-order predictive processes already take place in the preterm brain.

Evaluating predictive skills in neonates can be challenging but the tactile modality is a great candidate to explore sensory prediction in this population. Touch is the first sense to develop so it first allows the fetus to feel and be aware of its own body and the uterine environment. It is also the primary form of communication, a precursor of verbal communication, it is critical for bonding with the caregiver which is the foundation for healthy social behaviors and it is needed for the development of both gross and fine motor skills (Cascio, 2010). Preterm neonates already have the four peak complexes in EEG: P1, N2, P2, N3, typically seen during tactile stimulation of the adult hand (Whitehead et al., 2019). In the tactile modality, Dumont et al. (2022) recorded the neurovascular activity in the contralateral somatosensory cortex of preterm neonates born between 31 and 32 weeks GA during a stimulus omission paradigm using vibrotactile stimuli applied on the palm of the hand. The interstimulus interval could either be fixed (five seconds) or jittered (5 ± 3 seconds). As young as 33 weeks corrected GA, premature neonates had a strong RS with both fixed and jittered intervals, but after fewer trials in the fixed condition, suggesting quicker suppression in a less volatile environment, similar to what was found in infants several months old (Emberson et al., 2019). Changes during omissions also differed between fixed and jittered intervals. In the fixed condition, there was a significant decrease in blood flow index compared to baseline. Since the neurovascular response integrate all neural activity (both prediction and error signals), a decrease during omissions is not consistent with RS being the absence of an error signal, as this would not cause any change in brain activity. There seems to be an active inhibition of sensory processing beyond RS when the prediction is very precise. This mechanism has not been described elsewhere and more work is needed to understand its significance. On the contrary, omissions in the jittered condition were associated with a large increase in blood flow index, indicating that a prediction signal and possibly an error signal added up and resulted in a strong hemodynamic response. Results were interpreted as predictions with opposite regulatory (top-down) effects on the somatosensory cortex depending on initial bottom-up context volatility. As early as eight weeks before the term of pregnancy, expected stimuli in a highly predictable environment are suppressed, whereas partly expected stimuli in a moderately predictable environment yield marked anticipatory activity. In behavioral studies of attention, authors have proposed that moderate predictability of stimuli enhances attention allocated to their processing, whereas very high or very low predictability decreases attentional location. Learning would thus be facilitated by moderate predictability. For example, Téglás and Bonatti (2016) explored the conditions allowing 12-month-old infants to form predictions about future visual events. They found proactive expectation (as opposed to realizing post hoc that outcomes do not match with their previous experience, *i.e.,* a surprise effect) only when events were likely but not certain. Infants’ sensitivity to moderately predictable stimuli was also reported in term neonates (Bulf et al., 2011), and in 7- and 8-month-old infants (Kidd et al., 2012). Authors proposed that infants implicitly seek to maintain intermediate complexity of inputs, to avoid wasting cognitive resources on stimuli too complex or too simple. The results of Dumont et al. (2022) suggest this optimization is present before the age of term in the tactile modality. However, these results seem to contradict those from Emberson et al. (2017) and Boldin et al. (2018) describing impaired prediction in prematurely born infants, but several differences between studies may explain the apparent discrepancy. In Dumont et al. (2022), the population was born less premature and therefore might have less severe consequences of prematurity on sensory processing and prediction. Also, they were tested one week after birth, whereas Emberson et al. (2017) and Boldin et al. (2018) tested infants at six months of age: sensory prediction deficits could arise during post-natal development. Lastly, Emberson et al. (2017) and Boldin et al. (2018) tested the visual and auditory modalities. These develop during the last trimester of pregnancy and might have been more severely altered by untimely exposure to the extra-uterine environment than the tactile modality.

### 5.5 Neonatal prediction and neurodevelopment

More work is necessary to untangle the impact of modality, GA at birth and GA at measurement, on sensory prediction in premature neonates and other newborns at risk of ND, as well as to clarify the evolution of the various measures or predictive coding across the first months of development depending on birth history, and finally to provide evidence of their links with outcome. On the latter, Weber et al. (2016) recorded auditory ERPs during an oddball protocol at term-equivalent age in term-born or premature neonates born before 32 weeks GA. They investigated the “habituation” to the deviant stimulus, defined as the decrease of MMN amplitude along the presentation of the oddball sound, and showed that babies with the weakest “habituation” had lower development scores on Bayley’s scale at two years of age. They used an unusual stimulation paradigm that combines notions of “habituation” and MMN in a population where the mismatch response can vary in polarity and neural habituation is more generally measured as RS. Further investigations are needed to build on this finding using various paradigms and to determine which aspects of development are more specifically affected beyond what Bayley scores can inform, to allow for effective (*i.e.*, targeted) interventions. Schwarzlose et al. (2023) investigated the relationship between the BOLD response to unexpected auditory deviant stimuli at birth and behavioral inhibition at one year of age. They showed that newborns displaying larger responses during oddball stimuli in subcortical and temporal regions (which we can interpret as error signals) also had lower behavioral inhibition at one year old, whereas newborns with larger responses in prefrontal regions had higher behavioral inhibition at one year of age. Overall, the studies suggest that the activity and brain regions engaged during novelty detection and processing in perinatal development might pave the way to future skills critical for neurodevelopment, such as executive functions and autoregulation. This hypothesis, although it needs more experimental support, could become a major stake: if primary features of sensory processing at birth could predict neurodevelopmental outcomes, we would have leverage to bend developmental trajectories in more favorable ways. According to the neuroconstructivist framework, early sensory experiences and the construction of brain networks at the beginning of life constrain each other, thus guiding cognitive development toward a typical or atypical path (Karmiloff-Smith, 2009). Early interventions would be the most effective at minimizing impairment because they profit from the minimum specialization and maximum plasticity of the young brain (Johnson, 2011). Authors even proposed that interventions, when they have no adverse effects, should be proposed based on risk rather than evidence, because the period of maximum therapeutic effectiveness precedes clinical manifestations (Hendry et al., 2016). Therefore, proposing interventions targeting sensory prediction to infants at risk of ND may mitigate or even prevent symptoms.

### 5.6 Future directions in early neurodevelopment

Assuming that prediction and attention interact during child development and that they have a synergistic effect, facilitating each other’s function and development, atypical predictive abilities could interfere with attentional processes and vice versa. Atypical attention has been reported in ASD (Mutreja et al., 2016, Uddin et al., 2013) and proposed as a cross-syndromic feature of ND (Gargaro et al., 2011). The respective contributions of prediction and attention deficits need to be clarified to progress toward a unifying theory of ND. Although predictive accounts of ASD are promising because they provide a consistent explanation for most symptoms, they remain to be tested and compared with other theoretical propositions, and specific hypotheses with corresponding paradigms must be provided for this endeavor. A review of the links between sensory processing and attention, with emphasis on atypical neurodevelopment, can be found in Anquetil et al. (2022).

For both typically and atypically developing children, future work should aim at longitudinal and multimodal measures of more ecological stimulations, carefully distinguishing prediction and attention. The ecological aspect is particularly relevant for studying how prediction may be involved in atypical development, as children with a higher risk of ND tend to perform better when experimental conditions are less demanding. Unimodal oddball paradigms may thus not reflect the true extent of their deficits. Using complex, ecological, and even social stimuli to study the development of predictive coding in children is likely to be more fruitful in the long term. We also believe that experiments should be designed for diverse participants with and without ND, in order to include the analysis of the variability of brain responses among children and identify the factors of heterogeneity. Prediction and attention processes, as any experience-dependent mechanism, probably adapt to neurological, physiological, or environmental constraints along the course of development, and the resulting brain measures should reflect such adaptations. Describing the various trajectories of ERP components or BOLD responses to ecological predictive and attentional tasks is an ambitious yet critical goal for the field because it is a prerequisite for using these measures as clinically relevant biomarkers of ND risk at the individual level.

In children with ND, or children at high risk of ND such as infants born preterm or those who have a close relative with ND, a priority is to test the theoretical proposals of a high inflexible weight of error signals regardless of context reliability. Using various variability levels in background noise (*i.e.,* the “standard” stimuli stream) we can evaluate error signal amplitudes for local deviance processed by lower-order cortices. Using implicit statistical regularities in the sequence, we can evaluate error signal amplitudes for global deviance processed by higher-order cortices, and compare it with the same sequence when the global rule is provided explicitly. For ADHD, the underestimation of prediction precision may be better assessed using an omission protocol to study the amplitude of prediction signals in the absence of confounding activity. Omission paradigms are still under-used compared to the oddball, but they are complementary.

All this work should be made in several sensory modalities, including somatosensory, but also using intermodal paradigms. This would allow us to address the multiple types of impairments patients with ND may have. As we detailed in the “Prediction as an early screening marker” section, some etiological factors such as premature birth or exposure to teratogenic compounds may have modality-specific effects depending on the different sensitive periods of the senses. These factors likely have cascading effects on prediction in these sensory modalities.

Finally, we will want to tackle the role of sensory feedback of actions into our experimental paradigms. Indeed, besides sensory processing and attention, motor skills are the third functional domain affected across ND syndromes (McPhillips et al., 2014). Motor deficits appear early and are associated with later deficits in the acquisition of language (Bhat et al., 2012, Leonard et al., 2015), and even in typically developing children, motor development is related to statistical learning performance (Monroy et al., 2017). Interestingly, deficits in anticipatory posture adaptation and motor control have also been proposed as a pathological mechanism of autism (Schmitz et al., 2003). Predictive accounts of autism propose that repetitive behaviors would have the function of reducing input variability, thereby reducing error signals. But Supekar et al. (2021) showed that different types of repetitive behaviors are associated with different functional networks in the brain: cognitive control circuits (involving the salience, executive, and default-mode networks) for restricted interests and insistence on sameness, but motor circuits (cortical and subcortical motor networks) for repetitive motor actions such as rocking and hand flapping. The subcortical network consists of the basal ganglia and the cerebellum, the latter being considered the locus of efferent copies (*i.e.*, prediction) by proponents of the forward model (Sokolov et al., 2017). Future research will have to distinguish which theoretical proposal provides the best framework to explain ND symptoms and findings. In this process, predictive coding and forward model theories may evolve and even be combined to produce a new, embodied theory of predictive coding and its atypical development in ND. This process should take into account developmental changes in brain functions: at the beginning of brain development, before the primary motor cortex develops its motor functions, it works as a somatosensory area (Dooley and Blumberg, 2018). The switch between the sensory and motor functions occurs in rats at 12 days postnatal age, which corresponds to the human gestational full-term age (Romijn et al., 1991). The cerebellum also has protracted development, and motor control would therefore be built after birth, upon a sensory framework constrained mostly by prenatal experience and subcortical processes that remain to be elucidated. This could lead, for example, to neonatal or even prenatal sensory deficits being precursors of motor delays, a potential pathway to ND that has never been explored.

## 6 Conclusion

The predictive coding theory provides a framework to understand sensory processing and how it relates to cognition, but the complex hierarchical organization of sensory prediction in the adult brain is still being unraveled. Efforts should now focus on multimodal imaging approaches to refine the theoretical framework, understand the underlying brain mechanisms and their neural and hemodynamic measures.

In parallel, a few studies in patients with neurodevelopmental disorders found that their brain responses reflect atypical predictive processes from school age. Although deficits in prediction mechanisms are unlikely to explain the broad range of symptoms seen in ND, this highlights the relevance of the predictive coding framework to understanding core deficits in ND. More naturalistic and carefully crafted stimulation paradigms to control for attention, implicit rules and context reliability, are now required to test these hypotheses and their potential as early markers of ND risk.

Recent works show that predictive abilities are present in healthy children and infants, highly related to attention from an early age. This interaction likely plays a critical role in cognitive development, but we still lack data on toddlers and longitudinal studies are now crucial to understand how this system develops in typical and atypical trajectories, and how the corresponding brain measures of infants evolve into those of adults.

Given the potential for finding early ND biomarkers, recent studies have been performed in infants born preterm. They showed remarkably early predictive skills, that could be used as screening markers for ND risk, but also to help assess the effectiveness of preventive interventions before symptoms arise. As predictive coding overlaps with other theories of atypical neurodevelopment involving sensory prediction, in years to come we will aim to integrate these views in an embodied approach to gain a better picture of how predictive processes contribute to neurodevelopmental disorders.

## Funding

This research was supported by the NEOPRENE grant #ANR-19-CE37–0015 from the Agence Nationale de la Recherche (National Agency for Research) to NRL, and a Ph.D. grant from the French Ministry of Higher Education and Research to A-L.M.

## CRediT authorship contribution statement

**Marais Anne-Lise:** Writing – review & editing, Writing – original draft, Conceptualization. **Roche-Labarbe Nadege:** Writing – review & editing, Conceptualization.

## Declaration of Competing Interest

The authors declare that they have no known competing financial interests or personal relationships that could have appeared to influence the work reported in this paper.
